# Efeitos Anti-inflamatórios da Terapia com Atorvastatina na Síndrome Metabólica

**DOI:** 10.36660/abc.20210720

**Published:** 2021-10-06

**Authors:** Silvio A. Oliveira-Junior, Marianna R. Carvalho, Maria Lua M Mendonça, Paula F. Martinez

**Affiliations:** 1 Universidade Federal de Mato Grosso do Sul Campo GrandeMS Brasil Universidade Federal de Mato Grosso do Sul (UFMS), Campo Grande, MS - Brasil

**Keywords:** Síndrome Metabólica, Obesidade Abdominal, Diabetes Mellitus, Hipertensão, Endotélio/etiologia, Inflamação, Atorvastatina/tratamento farmacológico

A síndrome metabólica (SM) é caracterizada por obesidade abdominal, acompanhada de níveis elevados de glicose em jejum, resistência à insulina, hipertensão arterial e dislipidemia.^1^ Configura importante fator de risco para o desenvolvimento de outras condições clínicas, especialmente diabetes mellitus tipo 2 (DM2) e doenças cardiovasculares.^1 , 2^ Uma condição frequentemente relacionada à SM é a disfunção endotelial, caracterizada por alterações da arquitetura vascular arterial, sobretudo do endotélio e da membrana basal capilar, que ocasionam complicações microvasculares. Comumente, o remodelamento da membrana basal endotelial desencadeia processos de erosão e trombose vascular em indivíduos com distúrbios metabólicos.^2^

Com efeito, a patogênese da disfunção endotelial é multifatorial e pode incluir quadro crônico de inflamação, decorrente da ativação de leucócitos e aumento da produção de espécies reativas de oxigênio, eventos comuns na SM.^3^ O fenótipo pró-inflamatório derivado desta condição torna a vasculatura altamente vulnerável a processos inflamatórios com intensidade exacerbada, resultantes de mecanismos ativados, principalmente, por fator de necrose tumoral-alfa (TNF-α), proteína C reativa, interleucina-6 (IL-6) e interleucina-8 (IL-8).^4 , 5^ Em geral, altos níveis desses marcadores pró-inflamatórios configuram características comuns da SM.^5 - 7^ Além disso, a oxidação de lipoproteínas de baixa densidade (LDL) resultante de processos de peroxidação lipídica tem efeito imunogênico e pró-inflamatório, estimulando o recrutamento e concentração de células pró-inflamatórias.^5^ Por conseguinte, a disfunção endotelial se associa com proliferação de células musculares lisas e hipertrofia do tecido adiposo perivascular, características comuns ao processo de remodelação vascular.^8^

Classicamente, o excesso de gordura depositado ao redor de vasos e órgãos viscerais contribui para a instalação de quadro inflamatório crônico de baixo grau e resistência à insulina, desordens também comuns na obesidade.^9 , 10^ Nesse aspecto, o tecido adiposo perivascular tem importante função parácrina e está envolvido com a ativação de variados peptídeos que têm efeitos vasculares e contribuem para a disfunção endotelial comum na SM.^8 , 11^ Levando-se em conta as múltiplas respostas vasculares derivadas do aumento de adiposidade corporal e instalação de desordens metabólicas e cardiovasculares, torna-se essencial que estratégias terapêuticas sejam investigadas visando o controle desses eventos na SM. Nesse contexto, inibidores da hidroximetilglutaril coenzima A redutase (HMG-CoA), também chamadas estatinas, compõem um grupo de fármacos comumente utilizados para tratamento da doença cardiovascular aterosclerótica, com o princípio de controle de níveis séricos de colesterol.^12^ Entre as estatinas, a atorvastatina é atualmente uma das intervenções mais utilizadas no âmbito clínico, pois possui longa meia-vida plasmática e apresenta importante tolerabilidade e segurança.^13^

Na presente edição dos Arquivos Brasileiros de Cardiologia, Carvalho et al.,^14^ documentaram os efeitos do tratamento com atorvastatina sobre aspectos nutricionais, metabólicos e alterações vasculares em camundongos com SM induzida por dieta hiperglicídica. Como era esperado, a intervenção com dieta hiperglicídica resultou no aparecimento de diversos fatores associados com o desenvolvimento de SM. Os achados incluíram aumento da massa e adiposidade corporal, elevação nos níveis de colesterol total, lipoproteínas de baixa (LDL) e muito baixa (VLDL) densidades, hipertrigliceridemia, hiperglicemia e redução nos índices de lipoproteínas de alta densidade (HDL). Além disso, a dieta promoveu alterações no perfil inflamatório, com aumento nos níveis sistêmicos e teciduais de TNF-α e IL-6, hipertrofia do tecido adiposo perivascular e alterações vasculares.^14^ Na vigência de atorvastatina, os resultados confirmaram redução da massa e da adiposidade corporal e diminuição dos níveis de LDL, VLDL, triglicérides e aumento de HDL. No contexto morfológico, a atorvastatina reduziu a área seccional transversa e a espessura da camada média do vaso, concomitantemente à atenuação do tamanho e ao aumento no número de adipócitos perivasculares. Outro efeito importante da atorvastatina foi a redução dos níveis de TNF-α e IL-6 nos animais com SM, sugerindo que essas citocinas modulam o processo de remodelamento vascular.^15^

Portanto, os resultados deste trabalho mostram que intervenções com atorvastatina têm eficácia na prevenção e tratamento de condições cardiovasculares secundárias à SM. Novos estudos são necessários para elucidar potenciais mecanismos envolvidos com a instalação de desordens metabólicas e cardiovasculares na SM.
