# Supplementary material for: Efeitos Anti-inflamatórios da Terapia com Atorvastatina na Síndrome Metabólica
Source: Arq Bras Cardiol. 2021 Oct 6;117(4):748–9. [Article in Portuguese] doi: 10.36660/abc.20210720 (PMC8528359; doi:10.36660/abc.20210720)
Supplement: Translation

# Translation: Anti-Inflammatory Effects of Atorvastatin Therapy in Metabolic Syndrome

**Authors:** Silvio A. Oliveira-Junior, Marianna R. Carvalho, Maria Lua M Mendonça, Paula F. Martinez

**Affiliations:** 1 Brazil Federal University of Mato Grosso do Sul (UFMS), Campo Grande, MS - Brazil

**Keywords:** Metabolic Syndrome, Obesity, Abdominal, Diabetes Mellitus, Hypertension, Endothelium/etiology, Inflammation, Atorvastatin/drug therapy

Metabolic Syndrome (MS) is a condition characterized by abdominal obesity accompanied by high fasting glucose levels, insulin resistance, arterial hypertension, and dyslipidemia.^1^ It configures a critical risk factor for developing other clinical conditions, primarily type 2 diabetes mellitus (DM2) and cardiovascular diseases.^1 , 2^ Also, MS has been associated with endothelial dysfunction, characterized by changes in arterial vascular architecture, especially in the endothelium and capillary basement membrane, causing microvascular complications. Endothelial basement membrane remodeling commonly includes erosion and vascular thrombosis processes in individuals with MS.^2^

Endothelial dysfunction pathogenesis is a multifactorial event and may include chronic inflammation resulting from leukocytes activation and increased production of reactive oxygen species, which are commonly associated with MS.^3^ The pro-inflammatory phenotype related to MS makes the vasculature highly vulnerable to tumor necrosis factor-alpha (TNF-α), C-reactive protein, interleukin-6 (IL-6), and interleukin-8 (IL-8)-induced inflammatory processes.^4 , 5^ High levels of these pro-inflammatory markers are commonly associated with MS.^5 - 7^ Likewise, low-density lipoproteins (LDL) oxidation resulting from lipid peroxidation has immunogenic and pro-inflammatory effects, based on the recruitment and accumulation of pro-inflammatory cells.^5^ These effects may sustain endothelial dysfunction that is associated with smooth muscle cell proliferation and perivascular adipose tissue hypertrophy, events commonly observed during the endothelial remodeling process.^8^

Classically, ectopic fat deposition around visceral vessels and organs has contributed to low-grade chronic inflammatory conditions and insulin resistance onset, which is also common in obesity.^9 , 10^ In this aspect, perivascular adipose tissue has an essential paracrine function sustained by substantial protein activation and vascular morphological changes; these mechanisms contribute to the endothelial dysfunction during MS.^8 , 11^ Consequently, clarifying the potential efficiency of therapeutic interventions is essential to manage MS conditions. In this context, hydroxymethyl glutaryl coenzyme A reductase inhibitors (HMG-CoA), also called statins, are commonly used to treat atherosclerotic cardiovascular disease, and have proved efficacy in normalizing serum cholesterol levels.^12^ Indeed, atorvastatin is currently one of the most used statins in the clinical context as it has a long plasma half-life and presents significant tolerability and safety.^13^

In the current edition of the Arquivos Brasileiros de Cardiologia, Carvalho et al.^14^ documented beneficial effects of atorvastatin intervention on nutritional, metabolic and vascular aspects in an experimental model of hyperglycemic diet-induced metabolic syndrome. As expected, hyperglycidic diet-induced MS was accompanied by several disorders, as increased body mass and adiposity, high levels of total cholesterol, low (LDL) and very low (VLDL) densities hypertriglyceridemia, hyperglycemia, and reduced high-density lipoprotein (HDL) levels. Moreover, the dietary intervention promoted inflammatory effects, sustained by an increase in systemic and tissue levels of TNF-α and IL-6, perivascular adipose tissue hypertrophy and vascular changes.^14^ In response to atorvastatin, findings included a reduction in body mass and adiposity measures and decreased LDL, VLDL, triglycerides, as well as increased HDL levels. In the morphological context, atorvastatin reduced the transverse sectional area and the thickness of the vessel’s media layer and reduced the size and number of perivascular adipocytes. Another substantial atorvastatin effect included the reduction of TNF-α and IL-6 levels into MS, suggesting that these cytokines modulate the endothelial remodeling process.^15^

Therefore, the results of this study show that atorvastatin interventions may be effective as a medical intervention for preventive strategy and treatment of vascular conditions associated with MS. However, further studies are needed to elucidate potential mechanisms involved with metabolic and cardiovascular disorders in MS.
